# The selective orexin-2 antagonist seltorexant (JNJ-42847922/MIN-202) shows antidepressant and sleep-promoting effects in patients with major depressive disorder

**DOI:** 10.1038/s41398-019-0553-z

**Published:** 2019-09-03

**Authors:** Kasper Recourt, Peter de Boer, Rob Zuiker, Remy Luthringer, Justine Kent, Peter van der Ark, Ilse Van Hove, Joop van Gerven, Gabriel Jacobs, Luc van Nueten, Wayne Drevets

**Affiliations:** 1 Centre for Human Drug Research, Leiden, The Netherlands; 2 Leiden University Medical Center, Leiden, The Netherlands; 3 Janssen Research and Development, Division of Janssen Pharmaceutica N.V., Beerse, Belgium; 4 Minerva Neurosciences, Waltham, MA USA; 5 Sunovion, Fort Lee, NJ USA

**Keywords:** Clinical pharmacology, Pharmacodynamics

## Abstract

Excessive arousal has a role in the pathophysiology of major depressive disorder (MDD). Seltorexant (JNJ-42847922/MIN-202) is a selective antagonist of the human orexin-2 receptor (OX2R) that may normalize excessive arousal and thereby attenuate depressive symptoms. In this study, the effects of night-time arousal suppression on depressive symptoms were investigated. 47 MDD patients with a total Inventory of Depressive Symptomatology (IDS) score of ≥30 at screening were included in a randomized, double-blind, diphenhydramine-, and placebo-controlled multicentre study. Symptoms of depression were rated using the 17-item Hamilton Depression Rating Scale (HDRS_17_). Effects on sleep were evaluated by polysomnography and by the Leeds Sleep Evaluation Questionnaire (LSEQ). To investigate the safety and tolerability of seltorexant, vital signs, suicidal ideation and adverse events were monitored. At baseline the severity of depressive symptoms correlated with sleep efficiency (SE), wake after sleep onset (WASO), duration of stage 2 sleep, and ruminations. Ten days of treatment with seltorexant (and not diphenhydramine) resulted in a significant improvement of core depressive symptoms compared to placebo; the antidepressant efficacy of seltorexant was maintained with continued treatment up to 28 days. Compared to placebo, the antidepressant efficacy of seltorexant coincided with an overall increase in (left posterior) EEG power and a relative increase in delta- and decrease in theta-, alpha- and beta power during stage 2 sleep. Treatment with seltorexant was associated with mild, self-limiting adverse drug reactions. Seltorexant affected core symptoms of depression in the absence of overt changes in the hypnogram; in contrast, diphenhydramine was not efficacious.

## Introduction

In 2010 major depressive disorder (MDD) accounted for 8.2 percent of the global years lived with disability and 2.5 percent of the global disability-adjusted life years, thereby identifying MDD as a leading cause of the global burden of disease^1^. On a phenomenological level, certain MDD subpopulations demonstrate sustained negative affect such as subjective stress, anxiety and despair, cognitive phenomena, such as rumination^2^ and suicidal thoughts, various stress-related physical symptoms, and insomnia^3–7^. This hyperarousal that characterizes patients with MDD contributes to difficulties with falling asleep, staying asleep, and early morning awakening and may reflect a reduced ability to downregulate brain activity in limbic structures such as the amygdala during the sleep period^8^. This state of hyperarousal has been implicated mechanistically with hypothalamic–pituitary–adrenal (HPA)-axis hyperactivation^9–11^, and is supported by chronically elevated central nervous system (CNS) activation^12,13^ and, in certain MDD subtypes, sustained sympathetic nervous system activity^14^. Notably, the magnitude of abnormal HPA activity in depressed subjects versus controls is greatest during the night^14^. Failure to downregulate the arousal drive during the circadian nadir leads to neurobiological changes that contribute to depressive symptoms^15,16^. The neuropeptide orexin (OX) regulates arousal and wakefulness by modulating two distinct G-protein coupled receptors, orexin-1 (OX1R), and orexin-2 (OX2R). OX receptors are distributed throughout the brain and selectively expressed in certain brain structures that are involved in physiological responses to stress and panic^17^. Lateral hypothalamic orexinergic neurons have previously been demonstrated to be involved in maintaining wakefulness^18^ and show high neuronal activity during wake state while they are virtually silent during sleep^19,20^. OX activates both OX1R and OX2R during stress^21^, resulting in HPA activation which can be prevented selectively by OX2R antagonists^22^ and increased blood pressure and heart rate^23,24^, which can be prevented by OX1R antagonists^25^. In rodents OX antagonists ameliorated behavioral consequences of chronic unpredictable stress, normalized HPA axis function^26^, and attenuated an OX-induced increase in adrenocorticotropic hormone levels^27^. Despite promising preclinical evidence for a role of orexin-receptor antagonists in the treatment of depressive symptoms, at present clinical evidence is scarce. The dual OX1/2 (DORA) antagonist suvorexant is an approved treatment for insomnia^28^ but not for MDD. The dual receptor antagonist filorexant did not separate from placebo in a discontinued Phase II study in patients with MDD^29^. Insomnia, however, is an often occurring symptom during the course of depressive episodes and chronic insomnia and may be prodromal to MDD^30–33^. Studies of sleep architecture in MDD particularly document reduced capacity for deep sleep^34^. This finding suggests that DORAs may prove counterproductive in sleep disturbances associated with MDD, as preclinical data show that OX1R blockade in the presence of OX2R antagonism dysregulates rapid eye movement (REM) sleep^35^. However, as orexinergic neuron activity supports active wake during the diurnal activity cycle^36^ and preclinical studies in rodents suggest that the orexin-2 receptor (OX2R) is responsible for anti-arousal effects of OXR antagonism^37,38^, the current study aims to gather more clinical evidence for the effect of an OX2R antagonist in MDD patients. Although the basis of hyperarousal in MDD is unknown, we hypothesized that this phenomenon might relate to an inability to downregulate orexin activity. Seltorexant (JNJ-42847922/MIN-202) is a highly selective antagonist of the human orexin-2 receptor (OX2R) which is being developed as a treatment for major depressive disorder and insomnia. Seltorexant is CNS penetrant and demonstrates high in vitro affinity (pKi = 8.0) for the human OX2R with a 2 logs selectivity ratio versus the human OX1R^39^, making it a suitable candidate drug to specifically antagonize OX2R. Seltorexant was shown to improve sleep in patients with major depressive disorder (MDD) suffering from comorbid insomnia^40^. The selective histamine-1 receptor (H1R) diphenhydramine, was applied as a negative control because of its sedative properties and demonstrated lack of known mood-enhancing effects. This exploratory, safety, tolerability, and efficacy phase 1b study investigated the antidepressant effect of seltorexant in MDD patients. In addition, the effects of seltorexant on sleep stages and overnight EEG power spectra were evaluated. We reasoned that depression is associated with increased (negative) ruminations leading to an inability to initiate and maintain sleep because of a failure to downregulate the arousal drive during the circadian nadir. Overnight antagonism of the arousal drive by blocking OX2Rs was hypothesized to have benefit in the treatment of MDD.

## Subjects and methods

**Fig. 1:**
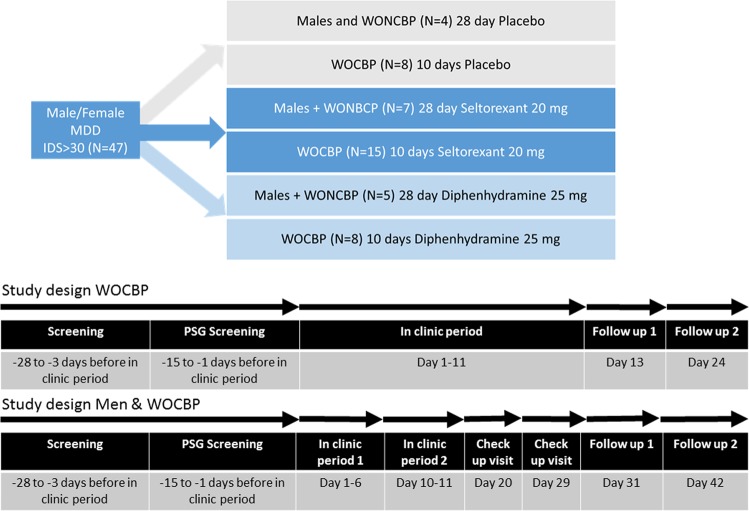
Study design and groups. The study consisted of a screening examination, including polysomnography (PSG) screening (day −28 to −1) at baseline, a parallel group treatment phase of 10 days (women of child-bearing potential [WOCBP]) or 28 days (women of non-child-bearing potential [WONCBP] and men), and a 2-week follow-up period. 47 patients with major depressive disorder (MDD) were randomized to seltorexant (*N* = 22), diphenhydramine (*N* = 13) or placebo (*N* = 12)

This was an exploratory double-blind, diphenhydramine- and placebo-controlled, multicentre study that took place in the Netherlands, Belgium, and Germany. Ethical and regulatory approval was obtained at each participating site/country by the following committees; Stichting Beoordeling Ethiek Biomedisch Onderzoek Assen, OLV Ziekenhuis Aalst, Vzw Emmaus Mechelen, UZ Brussel, Landesamt für Gesundheit und Soziales Berlin Geschäftsstelle der Ethik-Kommission des Landes Berlin, Ethikkommission der Ärztekammer Hamburg; Körperschaft des öffentlichen Rechts and Ethikkommission an der Medizinischen Fakultät der Universität Rostock. The study was registered on Clintrials.gov under NCT02476058. Informed consent was obtained from all subjects before any study activities took place. The study consisted of a screening examination (day −28 to −1) at baseline, a parallel group treatment phase of 10 days (women of child-bearing potential [WOCBP]) or 28 days (women of non-child-bearing potential [WONCBP] and men), and a 2-week follow-up period (Fig. 1). Owing to limited reproductive toxicology data at the time of the study, the duration of treatment was limited to 10 consecutive days for WOCBP. WOCBP were included because they constitute a large part of the target population in clinical practice and additional safety data in this population are important for future clinical studies. All WOCBP had a pregnancy test at screening and before admission to the clinic. All subjects were instructed to follow their normal daily routine when no study tests were scheduled. Subjects were randomized in a 2:1:1 ratio to receive seltorexant 20 mg, diphenhydramine 25 mg or placebo at bedtime. The subjects took one capsule daily from day 1 to 28 (day 10 for WOCBP) every evening just before bedtime with water. The capsules must be swallowed whole and not chewed, divided, dissolved, or crushed. In healthy subjects, doses ranging from 10 to 80 mg were considered safe^39^. The 20 mg dose for seltorexant was selected based on the predicted OXR2 occupancy, as well as the tolerability, and benefit/risk on sleep parameters established in previous studies^40–42^. From the phase I study program, it was clear that seltorexant at the dose used consistently induced sedation at a level that was expected to be approximated by diphenhydramine at a dose of 25 mg based on the summary of product characteristics. Because the appearance of diphenhydramine tablets was different from the seltorexant tablets, all tablets were over-encapsulated to maintain blinding and placebo was supplied as capsules containing neutral pellets. Assignment was based on a computer-generated randomization schedule prepared before the study. For each study site, the study pharmacist contacted the randomization center on day 1 before dosing after all inclusion and exclusion criteria had been met and confirmed by the study physician. Neither the investigator nor the pharmacist was provided with randomization codes. Patients aged 18–64 years with a body mass index (BMI) between 18 and 30 kg/m^2^, who met the Diagnostic and Statistical Manual of Mental Disorders, Fourth Edition, (DSM-IV) criteria for MDD without psychotic features and had a total Inventory of Depressive Symptomatology (IDS-C30) score of ≥30 at screening were recruited. The DSM-IV was used instead of the fifth version (DSM-V), because even though the DSM-V has been officially introduced in 2013, in practice, use of the DSM-IV was still favored by most of both GPs and psychiatrists during the time of study conduct. Patients with a current diagnosis of a psychotic disorder, MDD with psychosis, bipolar disorder, an eating disorder, mental retardation, cluster B personality disorder or a sleeping disorder were excluded. Patients were either antidepressant-naive or treated with a maximum of two concurrent monoaminergic antidepressants. The use of antipsychotic drug (D2-antagonists), lithium and other mood stabilizers or opiates was not allowed. Patients who had failed more than two pharmacological treatments with different modes of action prior to the current depressive episode, were excluded. Patients were tested for drug abuse during screening and at the start of every visit. The current and past diagnoses were confirmed by retrospective chart review and/or confirmation by their psychiatrist. Patients were required to be in acceptable physical health as determined at screening by medical history, physical examination, blood laboratory results and electrocardiogram. Symptoms of depression were evaluated using the 17-item Hamilton Depression Rating Scale (HDRS_17_)^43^. To establish the specificity of the antidepressant effect two HDRS_17_ subscales were used to explore the effect of sleep related items on the one hand (‘adjusted HRDS_17_’) and core items of depression on the other hand (‘HAM-D6’). The Sleep Item-Adjusted subscale is derived from the HDRS_17_ scale excluding the three insomnia questions from the total score. The six core symptoms of depression used in the HAM-D6 are: depressed mood, guilt feelings, work and interests, psychomotor retardation, psychic anxiety, and general somatics. The Inventory of Depressive Symptomatology (IDS-C30)^44^ was derived from the patient interview and the Quick Inventory of Depressive Symptoms (QIDS-SR16) completed by the patient. Rumination was assessed using the Ruminative Response Scale (RRS)^45^. Polysomnography (PSG) was performed on study days 1, 5, and 10. The PSG endpoints were latency to persistent sleep (LPS), total sleep time (TST), wake after sleep onset (WASO), wake within the total sleep period (WTSP), wake after final wakening (WAFA), time spent in deep sleep, and sleep efficiency (SE). Subjective sleep was scored using the Leeds Sleep Evaluation Questionnaire (LSEQ)^46^, a subject-reported 10-item visual analog scale (VAS) score used to rate quality of sleep. Additional unvalidated subjective assessment of sleep parameters were obtained in which subjects provided answers to ‘how long did it take you to fall asleep?’, ‘how long have you slept?’, and ‘how long were you awake after initial sleep onset?’ during last night to provide LPS, TST, and WASO estimates, respectively. PSG was recorded from electrodes C3-A2, C4-A1, F3-A2, F4-A1, O1-A2, and O2-A1 (A1 and A2 are left and right mastoid electrodes, respectively). EEG power spectra were calculated for the total non-REM period (N1, N2, and N3), separately for NREM N2 and N3, and for REM. EEG power spectra were derived by The Siesta Group (The Siesta Group Schlafanalyse GmbH, Wien, Austria) and contrasts versus placebo reported for total delta, theta, alpha, beta-1, -2, and -3 and gamma power reported (statistical threshold *p* < 0.10). Physical examination, body weight, supine vital signs, digital pulse oximetry, 12-lead electrocardiogram (ECG) and continuous ECG monitoring were performed and blood samples for serum chemistry and hematology and a urine sample for urinalysis were collected at baseline and throughout the study. Suicidal ideation using the Columbia Suicide Severity Rating Scale (C-SSRS)^47^ and adverse events (AE’s) were assessed locally by blinded staff. The study aimed to recruit 24 subjects in the seltorexant group, and 12 in both the diphenhydramine and placebo group. The study has been sized to obtain sufficient information on the safety of seltorexant during the observation period. For the exploratory endpoints, formal statistical calculations of sample size are not appropriate and descriptive statistics were used. The HDRS_17_ (and derived scales), IDS-C30, QIDS-SR16, RRS, subjective assessments of sleep parameters, LSEQ scores and any domain scores of interest were analyzed in a descriptive manner using frequency tabulations and summary statistics by treatment and timepoints. For the HDR_17_, IDS-C30 and QIDS-SR16, summary statistics were also produced excluding the insomnia questions from the total score (derived scales). The results of PSG parameters were tabulated by treatment, and summary statistics were calculated for each overnight PSG recording. Additional analysis was done for all patients, in which the correlation between HDRS_17_ and sleep-adjusted HDRS score and PSG-derived parameters was explored. In addition, the correlation (using univariate linear regression modeling) between the symptom severity of depression and rumination was explored as well as the relationship between rumination and sleep stages. Positive correlations (*p* < 0.05) in all patients were then further analyzed by comparing the change in the parameter of interest and change in HDRS_17_ score.

## Results

**Fig. 2:**
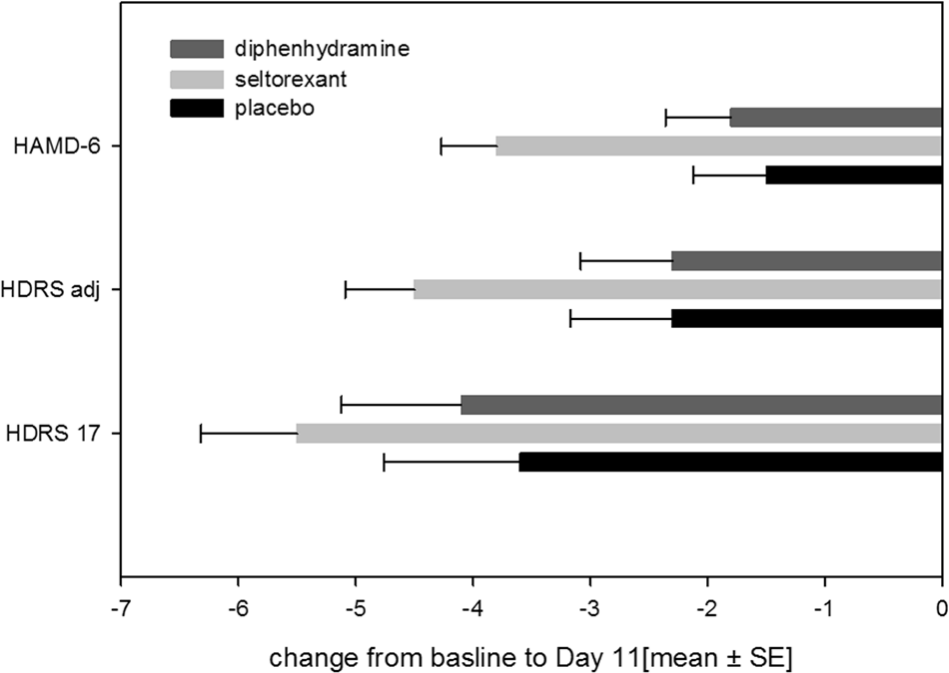
The antidepressant efficacy of placebo, seltorexant and diphenhydramine from day 1 (baseline) to day 11. Depressive symptoms were rated by the clinician using a structured interview. The HDRS_17_ total score was calculated as well as derived scales: HDRS-adjusted (sleep items removed) and HAMD-6 (core symptoms). An analysis using an ANCOVA model on the change from baseline with treatment and sex as factors and baseline score as covariate revealed a statistically significant larger reduction in the adjusted HDRS17 and HAMD-6 scores for seltorexant versus placebo (least-squares means difference −2.2, 95% CI [−4.35; −0.05], *F* = 4.37, *p* < 0.05 and least-squares means difference −2.5, 95% CI [−4.14; −0.80], *F* = 9.11, *p* < 0.01, respectively)

As is shown in the study disposition schedule (Fig. 1), a total of 47 subjects were randomized to seltorexant (*N* = 22), diphenhydramine (*N* = 13) or placebo (*N* = 12) during the period between 12-Jun-2015 and 04-Jan-2016. See Table 1 for a summary of the baseline characteristics. A total of 25 subjects continued the study as outpatients, receiving seltorexant (*N* = 12), diphenhydramine (*N* = 7) or placebo (*N* = 6) until day 28. Overall, the study population was characterized by a prolonged (>20 min) LPS (37/47 subjects [79 percent]). TST (<6.5 h) was reduced in 24/47 subjects [51 percent]. Most subjects were not currently treated with an antidepressant (10 of 47 [21 percent] subjects were taking an antidepressant of which 9 were treated with an SSRI and one with duloxetine). No differences in response to JNJ-42847922 were observed in subjects who received the compound as monotherapy compared to those who received it as an adjunctive therapy to a monoaminergic antidepressant drug. Seltorexant, compared to placebo and diphenhydramine, decreased the mean (SD) total HDR_17_ [−3.6 (4.03) and −4.1 (3.66) versus −5.5 (3.86), respectively]; also mean-adjusted HDRS_17_ [−2.3 (3.03) and −2.3 (2.81) versus −4.5 (2.76)] decreased more following seltorexant than placebo/diphenhydramine suggesting that the effect of seltorexant is not related to its effect on HDRS_17_ sleep items. Antidepressant effects of seltorexant unrelated to its effect on sleep items is also supported by a larger mean decrease in HAMD-6 [−1.5 (2.15) and −1.8 (2.01) versus −3.8 (2.22)] scores, a measure of core symptoms of depression, compared to baseline. Although the study was not powered to find a significant difference between the treatment groups, a post-hoc analysis using an ANCOVA model on the change from baseline with treatment and sex as factors and baseline score as covariate revealed a statistically significant larger reduction in the adjusted HDRS_17_ and HAMD-6 scores for seltorexant versus placebo (least-squares means difference −2.2, 95% CI [−4.35; −0.05], *F* = 4.37, *p* < 0.05 and least-squares means difference −2.5, 95% CI [−4.14; −0.80], *F* = 9.11, *p* < 0.01, respectively) (Fig. 2). Overall, the response to treatments measured on the HDRS_17_ was comparable in men and women from day 1 until 10 (Supplementary Fig. 1). In the outpatient group, significant efficacy of seltorexant versus placebo/diphenhydramine was maintained up to day 29 but only insofar core symptoms of depression (HAMD-6) were evaluated [*F*(2,24) = 4.133; *p* < 0.05] suggesting sustained efficacy of seltorexant; reductions in the HAMD-6 score on day 29 were −5.3 (3.45) and −2.0 (3.35) for seltorexant and placebo, respectively. No significant group differences were observed for change in total HDRS17 [*F*(2,24) = 1.665; *p* > 0.05] or sleep-adjusted HDRS [*F*(2.24) = 1.477; *p* > 0.05]. Self-reported depressive symptoms assessed by the QIDS-SR16 did not differ between the three treatment groups nor was there a significantly larger improvement measured in the seltorexant group versus placebo/diphenhydramine: The mean (SD) change from baseline in QIDS-SR16 total score at day 11 was −3.3 (4.12) in the placebo group, −3.5 (4.64) in the seltorexant group and −4.2 (4.17) in the diphenhydramine group [*F*(2,46) = 0.153; *p* > 0.05]. Thus, the clinician-rated improvement in depressive symptoms is not reflected in a patient-reported outcome measure. At baseline the total mean (SD) rumination scores per RSS were similar across treatment groups [55.6 (11.24), 53.6 (8.34), and 55.5 (10.10) for the placebo, seltorexant, and diphenhydramine groups, respectively]. Total RRS scores decreased following all treatments (−7.5, −8.6, and −5.1 for placebo, seltorexant, and diphenhydramine, respectively) compared to baseline [*F*(2,46) = 0.526; *p* > 0.05]. A numerical larger effect on the brooding subscale of the RRS was observed following seltorexant versus placebo from baseline to day 11 (−2.5 [2.61] versus −1.8 [3.66]) but treatments did not differ [*F*(2,46) = 0.924; *p* > 0.05]. The baseline total RRS, across all treatment groups, was significantly correlated with total HDRS_17_ scores [*R*^2^ = 0.223 and *F*(1,45) = 12.895; *p* < 0.01]; a similar, slightly tighter, relationship was observed after 10 days of treatment [*R*^2^ = 0.335 and *F*(1,44) = 24.211; *p* < 0.01]: therefore, higher total RRS scores were associated with more severe depression. While the brooding subscale of the RRS similarly correlated with depressive symptoms [*R*^2^ = 0.102 and *F*(1,45) = 5.13; *p* < 0.05] the relation was not observed for the reflection subscale [*R*^2^ = 0.058 and *F*(1,45) = 2.772; *p* > 0.05]. Improvements in HDRS_17_ were significantly correlated with a reduction in total rumination scores for seltorexant [*R*^2^ = 0.199 and *F*(1,20) = 4.954; *p* < 0.05] but not for placebo and diphenhydramine (Supplementary Fig. 2). Baseline LPS (mean [SD]) was numerically higher in the placebo-treated group than in either the seltorexant- or the diphenhydramine-treated groups (53.8 [40.12] versus 40.9 [22.63] and 36.0 [19.20] min) [*F*(2,44) = 0.621; *p* > 0.05]. Compared to placebo and diphenhydramine, there appeared to be a greater acute reduction in LPS following treatment with seltorexant (Supplementary Fig. 3); however, where the effect of seltorexant was sustained over time (day 1 versus day 11: −8.693 and −9.193 min [*t* = 0.049; *p* > 0.05]), the LPS effect following placebo increased over time (−3.375 versus −17.542 min [*t* = 0.709; *p* > 0.05]) while that of diphenhydramine decreased (−6.371 versus −0.269 min [*t* = 0.577; *p* > 0.05]). The acute and sustained effects on sleep induction were more clearly perceived by seltorexant-treated subjects by subjective report [mean (SD): −12.3 (18.72) and −11.1 (26.50) min] compared to baseline on days 1 and 11, respectively, versus 10.2 (37.71) and 1.0 (27.52) min and −2.12 (51.05) and 2.5 (38.49) min on days 1 and 11 following placebo and diphenhydramine, respectively, although differences between treatments are not significantly different (day 11 versus baseline: [*F*(2,44) = 0.0543; *p* > 0.05]). In line with the relative normalization of the LPS measured by polysomnography in all treatments, there were no consistent differences between treatments in their perceived ease of falling asleep. At baseline the mean TST did not differ across treatment groups [mean (SD)]: 376.4(31.82), 379.9(20.94), and 382.4(25.64) minutes for subjects randomized to placebo, seltorexant, and diphenhydramine, respectively ([*F*(2,44) = 0.0428; *p* > 0.05]; Table 2). Whereas LPS was overall prolonged in the study population, about 50 percent of the subjects had an objective TST < 6.5 h. There was no effect of treatment on TST from baseline to day 11; across all treatment groups TST tended to increase during the 10-day in-clinic period leading to a normalization of TST for most subjects (Table 2, Supplementary Fig. 4) [*F*(2,44) = 0.177; *p* > 0.05]. Also, PSG-derived WASO did not differ at baseline across treatment groups: 66.3 (21.10), 63.3 (16.93), and 68.8 (24.57) minutes for subjects randomized to placebo, seltorexant, and diphenhydramine, respectively [*F*(2,44) = 0.309; *p* > 0.05]. WASO measured overnight from day 10 to 11 was numerically reduced (compared to placebo) in diphenhydramine-treated subjects [32.5 (13.61) versus 51.9 (19.22) min] but not in seltorexant-treated subjects [45.2 (20.2) versus 51.9 (19.22) min]; treatment groups, however, did not differ significantly [*F*(2.44) = 1.215; *p* > 0.05]. Interestingly, a similar differentiation was not seen in subjectively reported WASO; in fact, subjects randomized to placebo reported the largest improvement. Treatment did not affect the number of arousals measured in the PSG (Table 3). Of the sleep parameters evaluated, sleep efficiency [*R*^2^ = 0.125 and *F*(1,45) = 6.433; *p* < 0.05], time spent in stage 2 sleep [*R*^2^ = 0.09 and *F*(1,45) = 0.09; *p* < 0.05], and wake after sleep onset [*R*^2^ = 0.100 and *F*(1,45) = 4.984; *p* < 0.05] at baseline were inversely correlated to the total HDRS_17_ score measured at baseline (Supplementary Fig. 5). WASO [*R*^2^ = 0.087 and *F*(1,45) = 4.287; *p* < 0.05] and duration of N2 [*R*^2^ = 0.092; *F*(1,45) = 4.569; *p* < 0.05] sleep, but not SE [*R*^2^ = 0.004 and *F*(1,45) = 0.193; *p* > 0.05], remained significantly related to the severity of depression when sleep items were subtracted from the HDRS17 suggesting that the relation between depressive symptoms and PSG-derived sleep parameters is not solely driven by HDRS_17_ sleep items. In contrast, LPS, duration of stage 1 and 3, and duration of REM sleep were not correlated to the baseline severity of depression. These relationships observed pre-dose were maintained across treatments on day 11. Improvements in HDRS_17_ did not correlate with the duration of stage 2 sleep and WASO. However, a trend towards a positive relationship was observed for SE in subjects treated with seltorexant [*R*^2^ = 0.136 and *F*(1,20) = 3.163; *p* = 0.09]. Power spectral analysis of the overnight sleep EEG showed that seltorexant, compared to placebo, tended to increase total power, predominantly left-sided spectral power (*p* < 0.10) while significantly increasing relative posterior delta power and decreasing posterior theta, alpha, and beta power (D10 versus baseline; Supplementary Fig. 6). LPS was not correlated with total RRS scores, however, duration of stage 1 sleep was associated with total RRS scores; more rumination was associated with longer stage 1 duration [*R*^2^ = 0.103 and *F*(1,45) = 5.149; *p* < 0.05]. Whereas duration of the stage 1 sleep correlated with the RRS total, sleep efficiency was not [*R*^2^ = 0.01; *F*(1,45) = 0.338; *p* > 0.05]. Treatment emergent adverse events (TEAEs) were reported separately for women of child-bearing potential (WOCBP) and males/postmenopausal and surgically sterilized women who received study medication for 11 and 28 days, respectively. A complete listing of the TEAEs can be found in supplementary table 1. Any significant changes in vitals, ECG, blood-, and urine analysis were reported as an TEAE. In the WOCBP group 4/6, 8/10, and 2/6 subjects reported TEAEs following placebo, seltorexant, and diphenhydramine treatment, respectively. These numbers were 3/6, 8/12, and 6/7 for placebo, seltorexant, and diphenhydramine, respectively, in male and female subjects who received study medication for 28 days. All TEAEs in the WOCBP group were reported by single subjects whereas somnolence and nasopharyngitis were reported by, respectively, 3 and 2 subjects who received seltorexant in the remaining subject group. The majority of the TEAE’s were mild to moderate in severity and all were transient. The suicidal ideation scores either improved or were maintained from screening through the end of study. One serious adverse event (SAE) was reported after completion of the study; one subject randomized to the diphenhydramine group completed suicide. No other SAEs occurred during the study

**Table 1:** Demographics and baseline characteristics

	Placebo (*N* = 12)	Seltorexant (*N* = 22)	Diphenhydramine (*N* = 13)
Demographics			
Age (years)	42.7 (12.66)	39.7 (13.98)	39.5 (12.13)
Gender female	4 (33.3%)	7 (31.8%)	5 (38.5%)
Gender male	8 (66.7%)	15 (68.2%)	8 (61.5%)
BMI (kg/m^2^)	23.7 (2.53)	24.9 (2.95)	24.1 (3.12)
IDS-C_30_			
Screening	38.3 (6.02)	37.7 (5.61)	40.5 (5.62)
Baseline	37.3 (6.79)	36.8 (5.84)	37.7 (8.99)
PSG parameters			
LPS (min)	53.8 (40.12)	40.9 (22.63)	36.0 (19.20)
TST (min)	376.4 (56.24)	379.9 (50.11)	382.4 (47.17)

Results are shown as mean values (SD); in addition, the absolute number and percentage of female and male subjects is shown

**Table 2:** The effect of placebo, seltorexant, and diphenhydramne on sleep maintenance

	Placebo			20 mg Seltorexant			25 mg Diphenhydramine		
PSG	Baseline	D1/2	D10/11	Baseline	D1/2	D10/11	Baseline	D1/2	D10/11
TST (min)	376.4 (31.82)	383.8 (43.39)	397.1 (23.96)	379.9 (20.94)	410.8 (19.31)	406.4 (21.26)	382.35 (25.64)	410.7 (23.54)	416.3 (20.09)
Diary									
TST (min)	334.2 (34.67)	335 (37.49)	365 (34.01)	341.8 (25.71)	379.6 (25.97)	367.5 (29.06)	348.1 (27.97)	345 (57.12)	361.2 (36.91)
PSG									
WASO (min)	66.3 (21.10)	62.3 (40.47)	51.9 (19.22)	63.3 (16.93)	46.3 (17.65)	45.2 (20.02)	68.8 (24.57)	47.7 (21.26)	32.5 (13.61)
aW (#)	21.6 (5.73)	19.3 (6.41)	17.4 (4.21)	18.2 (3.52)	16 (3.45)	17.3 (3.04)	19.5 (4.30)	18.5 (6.85)	15.4 (4.04)
Diary									
WASO (min)	59.2 (27.65)	63.3 (51.83)	25 (12.65)	55.1 (25.19)	40.3 (22.75)	31.8 (10.70)	61.9 (24.42)	73.5 (34.73)	45.8 (22.68)
aW (#)	3.7 (1.03)	4.5 (1.77)	2.8 (1.13)	3.7 (0.92)	3.5 (0.95)	3.5 (0.81)	4.2 (0.88)	2.9 (1.01)	3 (0.94)
LSEQ									
Fewer	NA	55 (10.55)	42.7 (8.51)	NA	42.8 (7.52)	41.9 (2.20)	NA	45.8 (7.39)	40.6 (7.52)

**Table 3:** The effect of placebo, seltorexant, and diphenhydramine on sleep induction

	Placebo			20 mg Seltorexant			25 mg Diphenhydramine		
PSG	Baseline	D1/2	D10/11	Baseline	D1/2	D10/11	Baseline	D1/2	D10/11
LPS (min)	53.8 (22.70)	50.4 (26.72)	36.2 (17.95)	40.9 (9.45)	32.2 (12.08)	31.7 (7.36)	36 (10.44)	29.3 (9.08)	35.8 (11.62)
Latency (min)	34.3 (15.12)	34.2 (21.33)	30.4 (17.27)	30.6 (9.52)	23.5 (6.73)	27.8 (6.45)	24.58 (9.23)	21.65 (6.72)	31.2 (11.69)
Diary									
Latency(min)	50.4 (18.98)	61.3 (21.98)	52.1 (21.90)	55.7 (15.85)	40.2 (9.04)	41.4 (8.56)	61.5 (22.15)	60.8 (31.32)	65.4 (24.04)
LSEQ^a^									
Easier	NA	55.1 (8.94)	41.9 (7.91)	NA	42.4 (7.43)	35.5 (8.55)	NA	44.9 (10.24)	46.1 (6.69)
Quicker	NA	55.9 (8.11)	43.6 (8.66)	NA	40.2 (7.91)	38.5 (8.58)	NA	47.8 (11.18)	47.5 (7.46)

Results are shown as mean values (95% confidence intervals)

^a^Getting to sleep easier/harder and getting to sleep quicker/slower

## Discussion

In this exploratory, safety, tolerability, and efficacy phase 1b study, we investigated the effect of seltorexant, a CNS penetrant and selective human OX2R antagonist, in both medicated and antidepressant-naive patients with MDD in a current major depressive episode of moderate severity. Although the basis of hyperarousal in MDD is unknown, we hypothesized that this phenomenon might relate to an inability to downregulate orexin activity^48^. We tested the effect of an OX2R-selective antagonist, seltorexant, because prior work in rodents had suggested that this receptor is responsible for anti-arousal effects of OXR antagonism^37^. That arousal-related sleep disturbances contributed to depression in our patient population is suggested by the significant correlation between sleep efficiency, time spent in stage 2 sleep, and wake after sleep onset with the total HDRS_17_ score and the HDRS score with sleep items subtracted measured at baseline. In contrast, LPS, duration of stage 1 and 3, and duration of REM sleep were not correlated to the baseline depression severity. The absence of an association between depression severity with LPS and duration of stage 1 sleep was unexpected given that hyperarousal is expected to impact the ability to enter stage 2 sleep. However, rumination was significantly associated with depression severity and although a relatively higher score on the RRS did not lead to a longer LPS, it was associated with an increase in the duration of stage 1 sleep. Altogether, these results suggest that depression severity is related to cognitive arousal and a more disrupted sleep profile. The improvement in self-reported depressive symptoms did not differ between the treatment groups, consistent with previous literature showing greater sensitivity of clinician-rated assessments to antidepressant effects (see below). However, a clinician reported antidepressant effect with early onset (as early as day 11 of exposure) of 20 mg seltorexant versus placebo and 25 mg diphenhydramine was demonstrated in the MDD sample irrespective of whether they received concomitant antidepressant therapy. This apparent contrast in clinician-rated and self-reported outcomes is reflected in meta-analyses that suggest that effect sizes based on self-report measures are smaller than those based on clinician-rated measures^49,50^. Although the reduction in the total HDRS_17_ score did not differ between treatment groups, the reduction on the HDRS_17_ score was statistically significant in the versions adjusted for sleep related items and on the six core symptoms of depression (HAMD-6). The study however, was not primarily powered to demonstrate an antidepressant effect, and since the population was only moderately depressed, as demonstrated by the average HDRS_17_ score of 19 at baseline, such limited changes could be the result of floor effects. This study was not designed to investigate sleep directly and MDD patients were selected without regard to insomnia symptoms. While seltorexant administration was not associated with significant changes in PSG parameters, it showed a trend towards improving patients’ self-reported sleep experience. Moreover, baseline TST and LPS measures correlated with the effect of seltorexant on these parameters suggesting that insomnia symptoms improved in subjects who entered the study with sleep disturbance. Similar discrepancies in improvements in subjective sleep quality versus sleep measured using polysomnography have been previously reported. Improvements in depressive symptoms following seltorexant (but not placebo and diphenhydramine) however, showed a nonsignificant trend toward a positive correlation with improvement in SE (*p* < 0.10); neither changes in WASO nor in duration of stage 2 sleep correlated with improvements in depressive symptoms in any treatment group. Within the construct of SE, the duration of stage 2 sleep was significantly associated with the severity of depressive symptoms at baseline. Power spectral analysis of the overnight sleep EEG showed that seltorexant, compared to placebo, tended to increase total, predominantly left-sided spectral power while significantly increasing relative posterior delta power and decreasing posterior theta, alpha, and beta power during stage 2 sleep. The association between depressive symptoms and PSG endpoints was found at baseline, post-treatment, and when sleep items were removed from the HDRS_17_. It may be hypothesized that the seltorexant-induced increase in delta power contributes to the antidepressant efficacy as delta power is relatively decreased in patients with MDD compared to controls and a similar pattern of EEG spectral changes was observed in a responder group to antidepressants. Orexinergic systems are silent during NREM sleep^19,20^ when recorded in rodents. If these systems work similarly in humans, the efficacy of an orexin antagonist overnight is enigmatic. Nevertheless, suvorexant, an FDA-approved sleep medication has been demonstrated to induce- and maintain sleep in insomnia patients. It may be hypothesized that although the orexin system is silent during NREM sleep, neurons resume activity during periods of arousal during sleep and that these arousal periods are more prominent in MDD patients. Seltorexant is hypothesized to antagonize these arousal periods thereby improving overall objective- and perceived sleep quality. Moreover, a 24 h CSF sampling study in MDD found elevated orexin release in depressed versus control subjects throughout the entire measurement period, along with a significantly blunted change in orexin levels across the circadian period^48^. Thus the OX2R antagonist mechanism may prove particularly helpful in depression to counteract the effect of hyperarousal via inappropriate orexin release during the night. Seltorexant 20 mg daily up to 28 days was safe and well-tolerated. The most commonly occurring AEs were somnolence, fatigue headache, dizziness, abdominal discomfort and nightmares and no SAEs occurred in the seltorexant treatment arm. The most extensive clinical safety data available on an orexin antagonist is for suvorexant, a dual orexin-receptor (OX1R/OX2R) antagonist (DORA), which was approved in the United States (US) and Japan in 2014 for the treatment of insomnia. Warnings for suvorexant include the risk of next day impairment, and symptoms similar to mild cataplexy. A contributing factor to these risks for suvorexant is its long half-life (about 12 h). In contrast, such risks appear to be mitigated with the relatively short half-life (2–3 h) of seltorexant and the absence of cataplexy as observed in the current study and in literature^40^. Overall, treatment with seltorexant showed antidepressant effects on core depressive symptoms as well as a trend towards improving patients’ self-reported sleep experience. This antidepressant effect with onset as early as day 11 of exposure of 20 mg seltorexant versus placebo and diphenhydramine in a relatively small number of patients with MDD warrants study in a larger sample of MDD patients.

## Supplementary information

Supplemental figure description

Supplemental table 1

Supplemental Figure 2

Supplemental Figure 3

Supplemental Figure 4

Supplemental Figure 5

Supplemental Figure 6
