# Correction to: The selective orexin-2 antagonist seltorexant (JNJ-42847922/MIN-202) shows antidepressant and sleep-promoting effects in patients with major depressive disorder

**DOI:** 10.1038/s41398-019-0585-4

**Published:** 2019-10-02

**Authors:** Kasper Recourt, Peter de Boer, Rob Zuiker, Remy Luthringer, Justine Kent, Peter van der Ark, Ilse Van Hove, Joop van Gerven, Gabriel Jacobs, Luc van Nueten, Wayne Drevets

**Affiliations:** 1 Centre for Human Drug Research, Leiden, The Netherlands; 2 Leiden University Medical Center, Leiden, The Netherlands; 3 Janssen Research and Development, Division of Janssen Pharmaceutica N.V., Beerse, Belgium; 4 Minerva Neurosciences, Waltham, MA USA; 5 Sunovion, Fort Lee, NJ USA

**Keywords:** Clinical pharmacology, Pharmacodynamics

**Correction to: Translational Psychiatry**

10.1038/s41398-019-0553-2 published online 3 September 2019

In the original Article, Tables two and three had formatting issues which affected their clarity. This has been corrected in the PDF and HTML versions of this Article.

