# Supplementary material for: The selective orexin-2 antagonist seltorexant (JNJ-42847922/MIN-202) shows antidepressant and sleep-promoting effects in patients with major depressive disorder
Source: Transl Psychiatry. 2019 Sep 3;9:216. doi: 10.1038/s41398-019-0553-z (PMC6722075; doi:10.1038/s41398-019-0553-z)
Supplement: Supplementary file 1 — Supplemental figure description [file 41398_2019_553_MOESM1_ESM.docx]

**Supplemental Figure 1: The antidepressant efficacy of placebo, seltorexant and diphenhydramine from Day 1 to Day 11 for males and females.**

On the x-axis the reduction in HDRS17 scores from baseline to Day 11 is shown; on the y-axis the proportion of subjects who minimally responded. Responses were bracketed in 10-percent intervals. Overall, the response to treatments measured on the HDRS_17_ was comparable in men and women.

**Supplemental Figure 2: Relationship between RRS total score and HDRS17**

The total RRS and HDRS17 are significantly correlated [R^2^ = 0.199 and F(1,20)= 4.954; *p*< 0.05] following 10 days of treatment with study medication. No significant correlation was found for placebo [R^2^ = 0.166 and F(1,10) = 1.993; *p*> 0.05] or diphenhydramine [R^2^ = 0.000 and F(1,11) = 0.042; *p*> 0.05].

**Supplemental Figure 3: The relation between the duration of LPS at baseline and LPS measured on Days 1, 5, and 10 .**

Dotted lines show the overall (on all study days) relation between baseline and post-dose LPS (per linear regression; not significant [F(2,44) = 0.0543; *p*> 0.05]). Open, light colored (closed) and dark colored (closed) circles represent Day 1, 5 and 10 data, respectively.

**Supplemental Figure 4: The relation between the duration of TST at baseline and TST measured on Days 1 and 10 .**

Dotted lines show the relation between baseline and post-dose TST on day 1 and 10 (per linear regression; not significant [F(2,44) = 0.177; *p*> 0.05]). Open and dark colored (closed) circles represent day 1 and 10 data, respectively.

**Supplemental Figure 5: Relationship between depression severity and PSG-derived sleep parameters at baseline**

The severity of depression was significantly related to sleep efficiency [F(1,45) = 6.433; *p*< 0.05], stage sleep duration [F(1,45) = 4.343; *p*< 0.05] and WASO [F(1,45) = 4.984; *p*< 0.05] such that higher sleep efficiency (Panel A) and stage 2 sleep duration (Panel B) were associated with lesser depressive symptoms while an increase in WASO (Panel C) was associated with an increase in depression. Green circles = placebo; blue circles = seltorexant; pink circles = diphenhydramine.

**Supplemental figure 6: Power spectral analysis of the overnight sleep EEG (day 10 versus baseline)**

Power spectral analysis showed that seltorexant, compared to placebo, tended to increase total power, predominantly left-sided spectral power (*p*< 0.10) while significantly increasing relative posterior delta power and decreasing posterior theta, alpha, and beta power (day 10 versus baseline)
