# Supplementary material for: The selective orexin-2 antagonist seltorexant (JNJ-42847922/MIN-202) shows antidepressant and sleep-promoting effects in patients with major depressive disorder
Source: Transl Psychiatry. 2019 Sep 3;9:216. doi: 10.1038/s41398-019-0553-z (PMC6722075; doi:10.1038/s41398-019-0553-z)
Supplement: Supplementary file 2 — Supplemental table 1 [file 41398_2019_553_MOESM2_ESM.docx]

**Supplementary table 1. treatment-emergent adverse events by body system or organ class and dictionary-derived term for males/WONCBP and WOCBP**

|  | **Males and WONCBP** | | | **WOCBP** | | | | **Total** |
| --- | --- | --- | --- | --- | --- | --- | --- | --- |
|  | Placebo  N=6 | Seltorexant  N=12 | Diphenhydramine  N=7 | Placebo  N=6 | Seltorexant  N=10 | Diphenhydramine  N=6 | N=47 | |
| **Body system**  Preferred term | N | N | N | N | N | N | N | |
| **Total no. Subjects with Adverse Events (%)** | 3 (50.0%) | 8 (66.7%) | 6 (85.7%) | 4 (66.7%) | 8 (80.0%) | 2 (33.3%) | 31 (66.0%) | |
| **Nervous System Disorders** | 2 | 5 | 4 | 0 | 2 | 1 | 14 | |
| Headache | 2 | 1 | 4 | 0 | 1 | 1 | 9 | |
| Dizziness | 0 | 1 | 2 | 0 | 1 | 0 | 4 | |
| Somnolence | 0 | 3 | 0 | 0 | 1 | 0 | 4 | |
| Migraine | 0 | 0 | 1 | 0 | 0 | 0 | 1 | |
| Syncope | 0 | 1 | 0 | 0 | 0 | 0 | 1 | |
| **Gastrointestinal Disorders** | 1 | 5 | 2 | 1 | 2 | 0 | 11 | |
| Abdominal Discomfort | 1 | 1 | 1 | 0 | 0 | 0 | 3 | |
| Abdominal distension | 0 | 0 | 0 | 1 | 0 | 0 | 1 | |
| Diarrhoea | 0 | 1 | 1 | 0 | 1 | 0 | 3 | |
| Nausea | 0 | 1 | 1 | 0 | 0 | 0 | 2 | |
| Abdominal Pain | 0 | 1 | 0 | 0 | 0 | 0 | 1 | |
| Abdominal Pain Lower | 0 | 1 | 0 | 0 | 0 | 0 | 1 | |
| Abdominal pain upper | 0 | 0 | 0 | 0 | 1 | 0 | 1 | |
| Dry Mouth | 0 | 1 | 0 | 0 | 0 | 0 | 1 | |
| Dyspepsia | 0 | 1 | 0 | 0 | 0 | 0 | 1 | |
| **Infections And Infestations** | 1 | 2 | 2 | 0 | 3 | 2 | 10 | |
| Nasopharyngitis | 0 | 2 | 1 | 0 | 1 | 1 | 5 | |
| Pharyngitis | 0 | 0 | 0 | 0 | 1 | 0 | 1 | |
| Gastroenteritis | 1 | 0 | 0 | 0 | 0 | 0 | 1 | |
| Influenza | 0 | 0 | 1 | 0 | 1 | 1 | 3 | |
| **General Disorders And Administration Site Conditions** | 0 | 2 | 2 | 2 | 2 | 0 | 8 | |
| Fatigue | 0 | 1 | 1 | 2 | 1 | 0 | 5 | |
| pyrexia | 0 | 0 | 0 | 0 | 1 | 0 | 1 | |
| Chest Discomfort | 0 | 1 | 0 | 0 | 0 | 0 | 1 | |
| Malaise | 0 | 0 | 1 | 0 | 0 | 0 | 1 | |
| **Psychiatric Disorders** | 0 | 2 | 2 | 2 | 1 | 1 | 8 | |
| Abnormal Dreams | 0 | 0 | 1 | 0 | 0 | 1 | 2 | |
| Abnormal sleep related event | 0 | 0 | 0 | 1 | 0 | 0 | 1 | |
| Completed Suicide | 0 | 0 | 1 | 0 | 0 | 0 | 1 | |
| Depression | 0 | 1 | 0 | 0 | 1 | 0 | 2 | |
| Insomnia | 0 | 1 | 0 | 1 | 1 | 0 | 3 | |
| Nightmare | 0 | 1 | 0 | 1 | 1 | 0 | 3 | |
| Anxiety | 0 | 0 | 0 | 0 | 1 | 0 | 1 | |
| apathy | 0 | 0 | 0 | 0 | 1 | 0 | 1 | |
| Restlessness | 0 | 0 | 1 | 0 | 0 | 0 | 1 | |
| **Eye Disorders** | 0 | 3 | 0 | 0 | 0 | 0 | 3 | |
| Blepharospasm | 0 | 1 | 0 | 0 | 0 | 0 | 1 | |
| Ocular Discomfort | 0 | 1 | 0 | 0 | 0 | 0 | 1 | |
| Vision Blurred | 0 | 1 | 0 | 0 | 0 | 0 | 1 | |
| **Respiratory, Thoracic And Mediastinal Disorders** | 1 | 0 | 2 | 0 | 1 | 0 | 4 | |
| Cough | 1 | 0 | 0 | 0 | 0 | 0 | 1 | |
| Dyspnoea | 0 | 0 | 0 | 0 | 1 | 0 | 1 | |
| Epistaxis | 0 | 0 | 1 | 0 | 0 | 0 | 1 | |
| Oropharyngeal Pain | 0 | 0 | 1 | 0 | 0 | 0 | 1 | |
| **Skin And Subcutaneous Tissue Disorders** | 1 | 1 | 0 | 1 | 0 | 0 | 3 | |
| Cold Sweat | 1 | 0 | 0 | 0 | 0 | 0 | 1 | |
| Sweat gland disorder | 0 | 0 | 0 | 1 | 0 | 0 | 1 | |
| Hyperhidrosis | 0 | 1 | 0 | 0 | 0 | 0 | 1 | |
| **Blood And Lymphatic System Disorders** | 0 | 0 | 1 | 0 | 0 | 0 | 1 | |
| Leukopenia | 0 | 0 | 1 | 0 | 0 | 0 | 1 | |
| **Cardiac Disorders** | 0 | 1 | 0 | 0 | 0 | 0 | 1 | |
| Palpitations | 0 | 1 | 0 | 0 | 0 | 0 | 1 | |
| **Injury, Poisoning And Procedural Complications** | 0 | 1 | 0 | 0 | 0 | 0 | 1 | |
| Laceration | 0 | 1 | 0 | 0 | 0 | 0 | 1 | |
| **Investigations** | 1 | 0 | 0 | 0 | 2 | 0 | 3 | |
| Alanine Aminotransferase Increased | 1 | 0 | 0 | 0 | 1 | 0 | 2 | |
| Aspartate Aminotransferase Increased | 1 | 0 | 0 | 0 | 1 | 0 | 2 | |
| Crystal Urine Present | 1 | 0 | 0 | 0 | 0 | 0 | 1 | |
| Lymphocyte Percentage Increased | 1 | 0 | 0 | 0 | 0 | 0 | 1 | |
| Urine Analysis Abnormal | 1 | 0 | 0 | 0 | 0 | 0 | 1 | |
| **Renal And Urinary Disorders** | 0 | 0 | 0 | 0 | 1 | 0 | 1 | |
| Dysuria | 0 | 0 | 0 | 0 | 1 | 0 | 1 | |
| **Reproductive system and breast disorders** | 0 | 0 | 0 | 0 | 1 | 0 | 1 | |
| Premenstrual headache | 0 | 0 | 0 | 0 | 1 | 0 | 1 | |
| **Metabolism And Nutrition Disorders** | 1 | 0 | 0 | 0 | 0 | 0 | 1 | |
| Hypophosphataemia | 1 | 0 | 0 | 0 | 0 | 0 | 1 | |
| **Musculoskeletal And Connective Tissue Disorders** | 0 | 0 | 1 | 0 | 4 | 0 | 5 | |
| Back Pain | 0 | 0 | 1 | 0 | 1 | 0 | 2 | |
| Arthralgia | 0 | 0 | 0 | 0 | 1 | 0 | 1 | |
| Musculoskeletal chest pain | 0 | 0 | 0 | 0 | 1 | 0 | 1 | |
| Pain in extremity | 0 | 0 | 0 | 0 | 1 | 0 | 1 | |
| **Vascular Disorders** | 0 | 0 | 1 | 0 | 0 | 0 | 1 | |
| Hot Flush | 0 | 0 | 1 | 0 | 0 | 0 | 1 | |

Note: Percentages calculated with the number of subjects in each group as denominator.

Note: Reported dictionary version: MedDRA 18.0
