# Supplementary figures and images for: The selective orexin-2 antagonist seltorexant (JNJ-42847922/MIN-202) shows antidepressant and sleep-promoting effects in patients with major depressive disorder
Source: Transl Psychiatry. 2019 Sep 3;9:216. doi: 10.1038/s41398-019-0553-z (PMC6722075; doi:10.1038/s41398-019-0553-z)

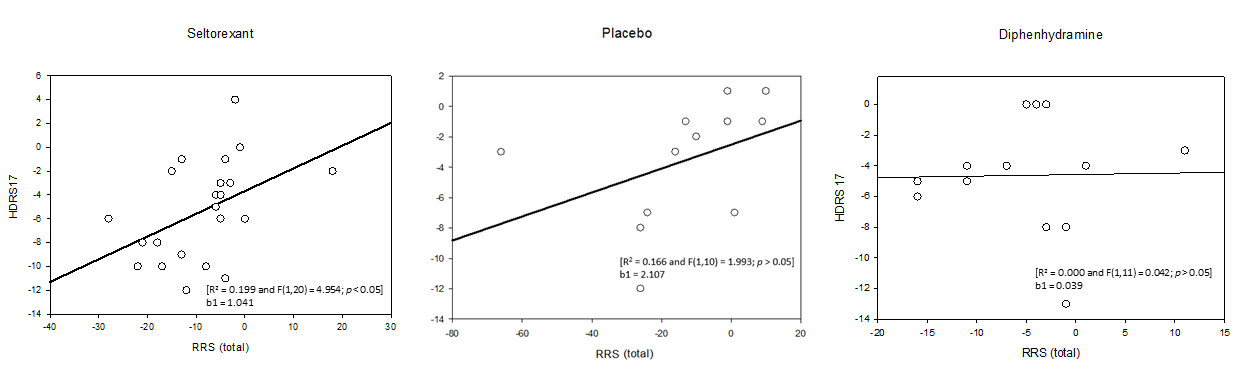

Supplement: Supplementary file 3 — Supplemental Figure 2 [file 41398_2019_553_MOESM3_ESM.tif]

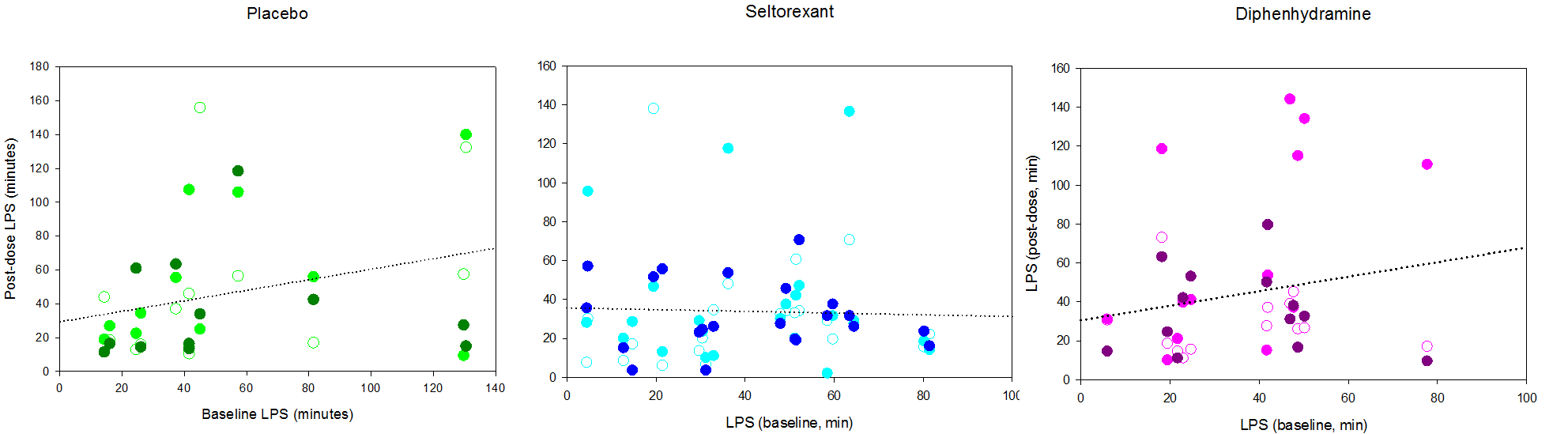

Supplement: Supplementary file 4 — Supplemental Figure 3 [file 41398_2019_553_MOESM4_ESM.tif]

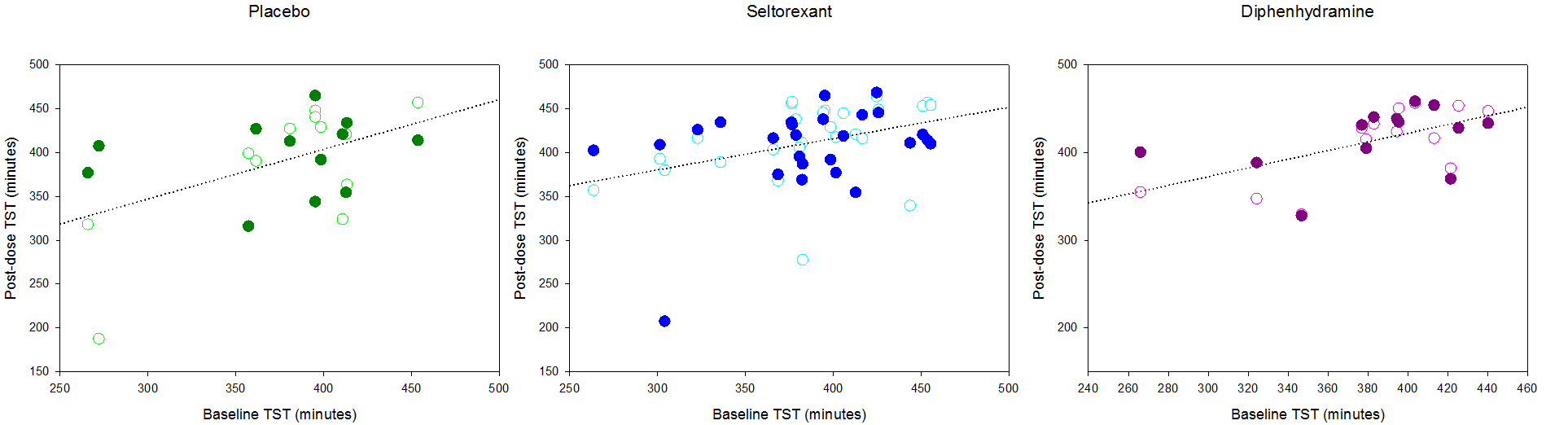

Supplement: Supplementary file 5 — Supplemental Figure 4 [file 41398_2019_553_MOESM5_ESM.tif]

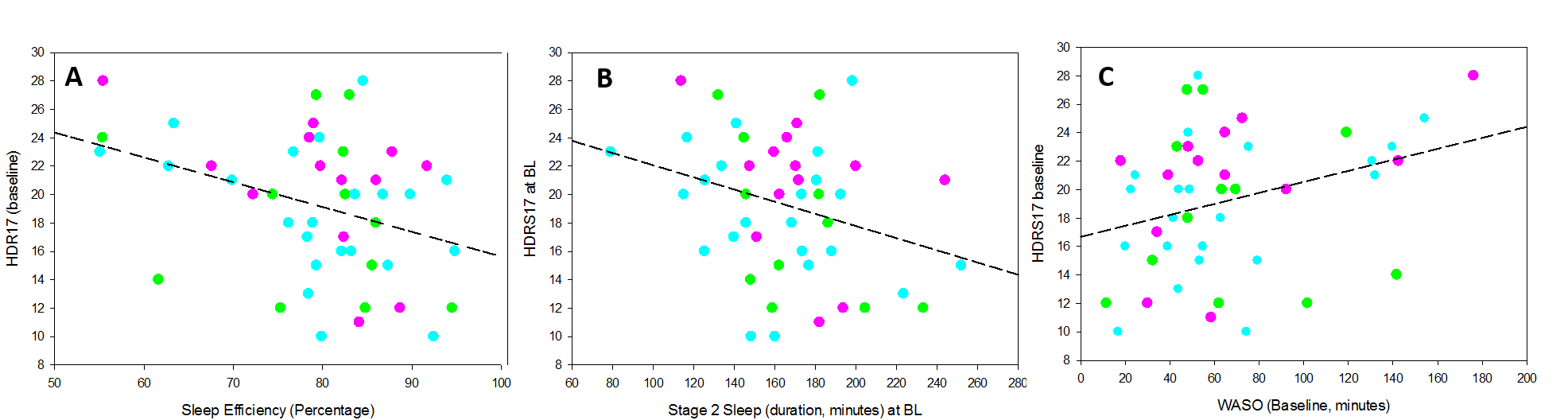

Supplement: Supplementary file 6 — Supplemental Figure 5 [file 41398_2019_553_MOESM6_ESM.tif]

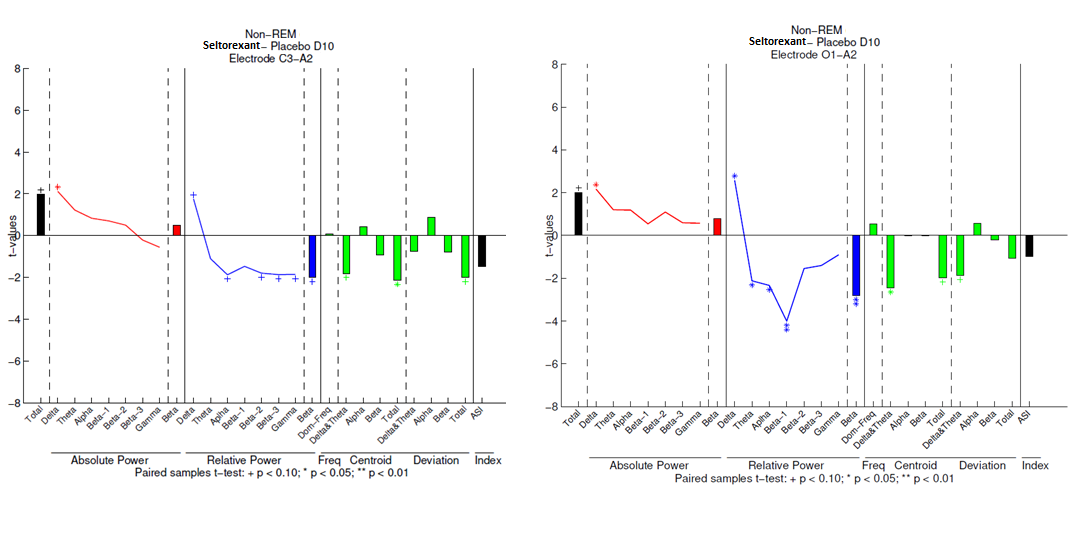

Supplement: Supplementary file 7 — Supplemental Figure 6 [file 41398_2019_553_MOESM7_ESM.tif]
